# A novel drug for uncomplicated malaria: targeted high throughput screening (HTS) against the type II NADH:ubiquinone oxidoreductase (PfNdh2) of *Plasmodium falciparum*

**DOI:** 10.1186/1475-2875-9-S2-I14

**Published:** 2010-10-20

**Authors:** Steve A Ward, Nicholas Fisher, Alasdair Hill, Alison Mbekeani, Alison Shone, Gemma Nixon, Paul Stocks, Peter Gibbons, Richard Amewu, David W Hong, Victoria Barton, Chandra Pidathala, James Chadwick, Louise Le Pensee, Ashley Warman, Raman Sharma, Neil G Berry, Paul M O'Neill, Giancarlo A Biagini

**Affiliations:** 1 Liverpool School of Tropical Medicine, Liverpool, L3 5QA, UK; 2 Department of Chemistry, University of Liverpool, Liverpool L69 7ZD, UK

## 

The mitochondrial respiratory chain of the malaria parasite *Plasmodium falciparum* differs from that of its human host in that it lacks a canonical protonmotive NADH:ubiquinone oxidoreductase (Complex I), containing instead a single sub-unit, non-protonmotive Ndh2, similar to that found in plant mitochondria, fungi and some bacteria [1,2]. As such, the *P. falciparum* Ndh 2 (PfNdh2) is a potentially attractive anti-malarial chemotherapeutic target. Using an E.coli NADH dehydrogenase knockout strain (ANN0222, *ndh::tet nuoB::nptI-sacRB*) we have developed a heterologous expression system for PfNdh2, facilitating its physicochemical and enzymological characterisation [2].

PfNdh2 represents a metabolic choke point in the respiratory chain of *P. falciparum* mitochondria and is the focus of a drug discovery programme towards the development of a novel therapy for uncomplicated malaria. Here we describe a miniaturised spectrophotometric assay for recombinant PfNdh2 (steady state NADH oxidation and ubiquinone reduction monitored at 340 nm and 283 nm respectively) with robust assay performance measures that has been utilised for the high throughput screening (HTS) of small molecule inhibitors.

The objectives of the HTS were twofold: (i) Increase the number of selective PfNdh2 inhibitors and (ii) to expand the number of inhibitor chemotypes. At the time of screening, only one proof of concept molecule, 1-hydroxy-2-dodecyl-4-(1H)quinolone (HDQ), was known to have PfNdh2 inhibitory activity (IC50=70 nM) [3,4]. HDQ was used to initiate a primary similarity-based screen of 1000 compounds from a compound collection of 750,000 compounds (curated by Biofocus-DPI). Chemoinformatics methodology was applied to the hits from this initial phase in order to perform a hit expansion screen on a further ~16,000 compounds. Application of this chemoinformatic strategy allowed us to cover ~16% diversity whilst screening just ~2% of the compound collection.

The HTS resulted in a hit rate of 0.29% and 1 50 compounds were progressed for potency against PfNdh2. Of these compounds, 50 were considered active with IC50s ranging from 100 nM to 40 μM. Currently seven distinct chemotypes are being progressed from hit to lead using traditional synthetic medicinal chemistry strategies.
